# Central Auditory Processing Disorders in Individuals with Autism Spectrum Disorders

**DOI:** 10.4274/balkanmedj.2018.0853

**Published:** 2018-09-21

**Authors:** Emre Ocak, Rebecca S. Eshraghi, Ali Danesh, Rahul Mittal, Adrien A. Eshraghi

**Affiliations:** 1 Department of Otolaryngology and Neurological Surgery, University of Miami Miller School of Medicine, Miami, USA; 2 Department of Gastroenterology, University of Miami Miller School of Medicine, Miami USA; 3 Department of Audiology, Florida Atlantic University School of Medicine, Miami, USA

**Keywords:** Autistic disorder, auditory processing disorder, central auditory processing, mismatch negativity, P300

## Abstract

The etiology and the underlying pathogenetic mechanisms of autism spectrum disorders are still largely unknown. This article provides a comprehensive review of the studies that are relevant to autism spectrum disorders and central auditory processing disorders and also discusses the relationship between autism spectrum disorders and central auditory processing disorders in the light of recent studies on this subject, which may provide new pathways in a therapeutic perspective. Several studies confirm that most of the individuals with an autism spectrum disorder have some degree of sensory dysfunction related to disorders of processing auditory, visual, vestibular, and/or tactile stimuli. Among these studies, some have addressed central auditory processing disorders. There is an increasing amount of effort for studies regarding the link between autism spectrum disorders and central auditory processing disorders. Most of the studies investigating central auditory processing disorders in patients with autism spectrum disorders have used electrophysiological measurements such as mismatch negativity and P300 event-related potentials. In addition to these, several studies have reported deterioration in speech perception and expression in patients with autism spectrum disorders, which may also be related to central auditory processing disorders in this unique group of individuals.

Autism spectrum disorder (ASD) is a heterogeneous group of disorders with an estimated prevalence of approximately 14.7 per 1000 among children aged 8 years. Abnormal repetitive and stereotypic behaviors, restricted interests in early childhood, and deficiency of social communication are the characteristic clinical features of ASD, and boys are more likely to be affected than girls (1,2). According to the Diagnostic and Statistical Manual of Mental Disorders (DSM-IV)-text revision, the disease was subcategorized within the diagnosis of “pervasive developmental disorders (PDD).” As this definition was open to argument, the more recent DSM-V made it simpler and gathered different diagnoses (autistic disorder, Rett syndrome, Asperger’s syndrome, childhood disintegrative disorder, and PDD not otherwise specified) together to generate the diagnosis of ASD (3). The etiology and the underlying pathogenetic mechanisms are still largely unknown, but studies show that complex environmental factors in conjunction with genetic factors play an important role in the etiogenesis (4,5). Having a sibling with ASD, having older parents (a mother who was 35 years or older, and/or a father who was 40 years or older when the baby was born), and carrying certain genetic conditions such as fragile-X syndrome, Down syndrome, and tuberous sclerosis are the most widely studied factors. Several studies confirm that most of the children with ASDs have some degree of sensory dysfunction (6). This may be related to disorders of processing auditory, visual, vestibular, and/or tactile stimuli. Among these, a recent study addressed central auditory processing disorders (CAPDs) as the most severely affected in ASD (7). CAPDs have been an underestimated issue in ASDs for a long time. However, researchers are now becoming aware of their significance and impact on autism. It is now time to focus on how CAPDs are related to ASDs and their potential consequences on treatment modalities. In this regard, the aim of this article is to review the relationship between ASDs and CAPDs in the light of recent studies on the subject.

## CENTRAL AUDITORY PROCESSING DISORDERS AND AUTISM SPECTRUM DISORDERS

**Figure 1:**
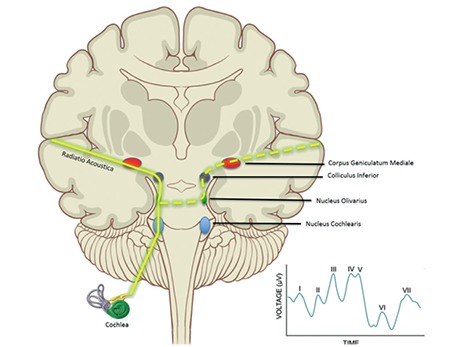
Overview of the central auditory system. Dotted yellow line represents the contralateral stimulation of the auditory cortex as the sound received from one cochlea stimulates the auditory cortex bilaterally. The graph represents the waves acquired by the auditory brainstem and the cortical responses. Each wave demonstrates the integrity of different components of the auditory system.

**Figure 2:**
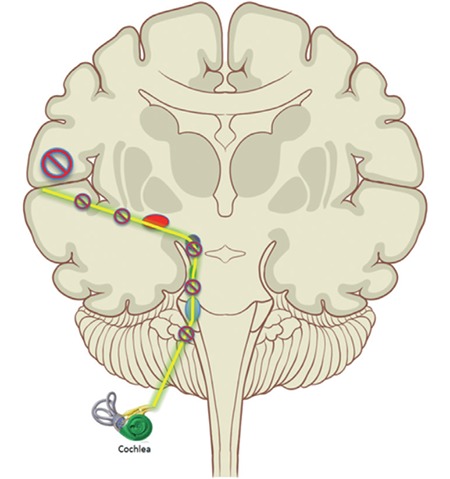
Blockage at any part of the transmission route may cause central auditory processing disorders.

In the early 1950s, Mykelbust suggested that children with language disorders have an auditory deficit even if they have normal functioning peripheral hearing (8,9). This was the first known step on the subject of auditory processing. However, since there were no audiologic tests to determine central auditory functioning, no further studies were performed on this issue. For a long period of time Mykelbust’s statement stood just as an idea. Later on, some researchers published their results supporting the idea that some patients may have difficulty in retrieving, analyzing, transforming, organizing, and storing information despite an intact peripheral hearing system and audible signals (10,11,12). After numerous studies began enlightening the dark pages of the central auditory system, CAPD was first officially described in 1992 by the American Speech–Language–Hearing Association (Figure 1 and 2) (13).

There is an increasing amount of effort for studies regarding the link between neurodevelopmental disorders and CAPDs. Diagnosis of CAPD is not easy, and a multidisciplinary approach between, but not limited to, speech-language pathologists, audiologists, psychologists, and neuroscientists focusing on the patients’ auditory, learning, and language characteristics is critical. Yet the true diagnosis is difficult to be made in most of the times, particularly in early childhood. Both electrophysiological and behavioral tests are recommended to be performed in combination. Behavioral tests provide a great amount of important information regarding the different components of the central auditory system. With regard to neuromaturation and neuroplasticity of the auditory system, some behavioral tests have limited place for use in children below the age of 7 years. This is because of the progressing neuromaturative development of some components of the auditory system until age 12 years or even later. In cases where the patient is too young to perform adequately in behavioral tests, electrophysiologic measures become more prominent. These tests are recordings of electrical potentials that reflect the synchronous activity generated by the central nervous system in response to different acoustic stimuli. The recordings of the cortical brain potentials during information processing can be represented as event-related potentials (ERP). ERPs can be extracted from electroencephalogram data. The ERPs in the context of the central auditory system can be subcategorized, such as auditory brainstem responses, steady-state evoked potentials, middle latency responses, mismatch negativity (MMN), and cortical ERPs (P1, N1, P2, P300), according to the anatomical region and the time of recordings after the stimulus. As more studies on CAPDs emerged, some models have been designed and proposed for better understanding the situation. Bellis/Ferre and Buffalo are the two most popular and widely accepted CAPD models in the literature. Both these models have subcategories according to the place and features of the deficit. In the Buffalo model, these categories consist of decoding, tolerance fading memory, integration, and organization. In the Bellis/Ferre model, there are three primary subprofiles consisting of auditory decoding deficit, prosodic deficit, and integration deficit. Although ASDs may be related to more than one category, a pioneer in the field and one of the founders of CAPD models, Bellis (14), stated that CAPD does not cause disorders such as ASD and the impairments seen in the central auditory system of patients with ASDs should be considered as associated symptoms. As arguments concerning the role of CAPDs in ASDs have been ongoing, several electrophysiological studies indicated that CAPD is actually one of the primary characteristic features of ASD.

### Electrophysiological Studies

Most of the studies investigating CAPDs in patients with ASDs used electrophysiological measurements such as MMN and P300 wave morphology. MMN is the response of the brain to a deviant stimulus within a sequence of otherwise regular stimuli. This deviance of stimuli can be in either intensity, frequency, or duration. MMN reflects the automatic detection of an acoustic stimulus change. In a study investigating auditory change detection in children with ASD, the MMN responses of 11 children with ASDs were compared to those of 11 children with typical development. The responses were significantly reduced in children with ASDs compared to those in controls in terms of both words and pseudowords regarding a pathology in the frontal regions of the brain (15). Another study suggested that autistic school-age children do have auditory changes at the level measured by MMN, primarily preattentive responses (16). Vlaskamp et al. (17) investigated MMN and P3a amplitudes in a large sample of children. The results indicated that MMN responses were reduced and P3a amplitudes were increased significantly in patients with ASDs. Shortened latencies and reduced amplitudes in MMN responses to different deviant stimuli were reported in several other studies (18,19,20,21,22,23,24,25,26). The P300 is a late positive component of an ERP, with a latency of approximately 300 ms after the presentation of a stimulus. It is an endogenous potential expressing the electrical activity of the brain associated with anticipation of the stimulus and is usually recorded from midline electrodes. The oddball paradigm is a discrimination task that is used in P300 recording. It consists of a series of randomly administered untargeted and targeted stimuli. The P300 wave is described using latency and amplitude and can be used as an indicator of several neuropsychological pathologies (27).

Abnormalities in P300 wave amplitudes and latencies have been reported previously in neurodevelopmental and neurophysciatric disorders (28,29). In a recently published meta-analysis, data from 32 studies were obtained. A total number of 407 patients with ASDs were compared with 457 controls with typical development, and the results revealed that patients with ASDs have impairment in the P300 component of ERPs (30). Another study by Ishikawa et al. (31) suggested that autistic features can affect the P300 responses to unexpected conditions. The results of this study are interpreted as a cause of decreased availability of context information. The recently published clinical studies on ASDs using electrophysiological tests are summarized in Table 1.

### Speech Perception and Expression in Autistic Patients

Communication and language impairment is indicated as one of the core characteristics of ASDs. Several publications have reported deterioration in speech perception in patients with ASDs. It has been advocated to be a component of the global sensory deficit (32,33,34). One interesting hypothesis regarding this issue is the integration deficit of the auditory and visual speech information. Tryfon et al. (35) reported that the diminished capacity of autistic children to integrate sensory inputs across visual and auditory systems causes impairment in speech perception, especially in noisy environments where the visual signals, such as seeing the speaker’s mouth, enhance the ability of speech perception.

Although theories about the speech perception impairment have been discussed in several reports, there are very limited data regarding the speech expression deficits in patients with ASDs. A recent study advocated that the impairment of the speech output may be a primary deficit (36). That study reported that infants at high risk for autism produce fewer speech-like vocalizations and syllable shapes than those produced by the neurotypical infants. Another study by Peeva et al. (37) investigated the left ventral premotor cortex (vPMC), which plays an important role in the generation of speech expression and is an important component of speech motor planning and network of areas involved in speech production, including supplementary motor area (SMA), ventral motor cortex, and posterior superior temporal gyrus. Their results revealed weaker connection between the vPMC and the SMA in the population with ASDs (37). Hearing rehabilitation of patients with ASDs who have severe sensorineural hearing loss can be achieved with cochlear implantation. There are limited data about the outcomes of cochlear implantation in patients with ASDs (38). After the implantation, the speech outcome can be affected because of the dual diagnoses in these patients. In a study regarding cochlear implantation in autistic individuals, Eshraghi et al. (39) proposed new assessment systems for speech perception and expression scales as existing evaluation parameters would not be appropriate to assess this unique group of patients. These new scales allow to assess easily, by caregivers, the function of speech and the perception of expression. They also help to observe progress over time and after interventions without the need of an extended battery of questionnaires. There is suspicion that the impairment of speech perception may be due to CAPDs, but how about speech expression? Can the apraxia of speech in this population be related to CAPDs? Although there are very limited data in the literature, the possibility of the connection of these impairments as a part of CAPD is a high probability. There is a need for further research in this field to provide more scientific evidence.

## DISCUSSION

Previous studies demonstrate that the development of multiple regions of the brain such as the cerebellum, the amygdala, and the frontal cortex is impaired in patients with ASDs (40,41,42,43). In addition, several morphological and radiological studies have reported consistent alterations in the hippocampus, the fusiform gyrus, and the frontoinsular and cingulate cortices (41,42,43). Patients with ASDs may have cognitive, attentional, and working memory processing deficiencies. However, it is still unclear whether the working memory dysfunction in patients with ASDs occurs during decision-making phases. These findings may suggest a general delay in the speed of information processing. Focusing on the central auditory system, several studies have also documented delays in auditory processing in ASDs, but there is an obscurity whether these are evidence of a CAPD specific to an ASD (44,45,46). Several studies can be found in the literature, which are controversial with each other on the issue. For instance, some studies suggest reduced processing speed of stimuli in ASDs (47,48), whereas some fail to identify any difference between patients with ASDs and controls (49,50). It is difficult to perform behavioral tests in autistic patients as they hardly accommodate the instructions. Thus, electrophysiological studies play an important role in the diagnosis of these individuals. In this regard, numerous studies have been performed, demonstrating impairment in the central auditory system in ASDs (16,17,18,19,20,21,22). In addition to these, there are some neuroimaging studies such as those using functional magnetic resonance imaging and positron emission tomography, demonstrating less activation by complex sounds in autistic children in Brodmann’s areas 21 and 39, which are thought to be auditory associative and involved in word processing. These studies indicate that in patients with ASDs, there may be a dysfunction of specific temporal regions, which have an important role in perception and the integration of complex sounds (51,52). Studies also provide histopathological evidence of a consistent and significant decrease in the number of superior olivary complex neurons in the autistic brain, which suggests a possible pathology at the brainstem level (53). One can expect that the presence of CAPD will impact multiple areas in these patients in relation to communication, sensory problems, and even balance. Another important issue is the outcomes of patients with ASDs after cochlear implantation. Limited number of studies about cochlear implantation in patients with an ASD reported satisfactory hearing outcomes, but implanted patients with ASDs still do not usually develop the expected language skills compared to those with normal neurodevelopment (39,54). Scientific data from ERP studies reveal that despite normal basic sensory perception, patients with ASD may have abnormal cognitive processing of auditory information, and this can explain the insufficient outcomes of language skills in this unique group of patients (55,56,57,58). Moreover, studies reveal that traditional audiological assessments may be insufficient for implanted patients with ASD as they should be evaluated with more specific tests. In this regard, the authors promote more practical and functional outcome measures to assess these patients who have received a cochlear implant. Once CAPD is diagnosed, a team approach, including a speech-language pathologist, an audiologist, teachers, and parents, should be utilized for the treatment. The approach should be individualized for each patient based on several variables such as preferred language, cultural background, and communication needs. The American Speech–Language–Hearing Association recommends three treatment approaches that are often used concurrently. These are “direct skills remediation” such as computer-based training, “compensatory strategies” such as the recruitment of higher order skills, and “environmental modifications” (59,60,61). Not limited to the auditory system, there is a global impairment for sensation in patients with ASDs (3). Several abnormal behavioral manifestations can be related to impairments in the central processing system. The underlying mechanism of repetitive behaviors may include covering the absence of sensation. It has been reported that patients with ASDs desire for pressure on their skin most probably just for additional stimulation to help compensate for the overall lack of other sensory inputs such as proprioception. For instance, weighted vests are recommended for children with ASDs (62). Moreover, some studies indicate that weighted blankets can improve sleep in children with ASDs (63). Despite numerous studies in the field, further research is needed on the subject to clarify the underlying mechanisms related to ASDs and understand the affected areas in the central auditory system and the role of CAPDs. Although there is not enough evidence to suggest that patients with ASDs are at a greater risk of hearing loss than the normal population, there is strong evidence obtained from electrophysiological and behavioral tests indicating the presence of CAPDs in individuals with ASDs. However, it is still unclear to conclude that CAPD is the primary contributing factor for communication disorders in these patients.The impact of CAPDs in patients with ASDs is not well recognized. This is related most probably to the complexity of factors affecting different regions of the brain. As the behavioral components of ASDs can also be related to CAPDs, understanding this complex connection will lead to finding the most accurate therapeutic approaches to help increase the quality of life of these individuals.

## Figures and Tables

**Table 1:**
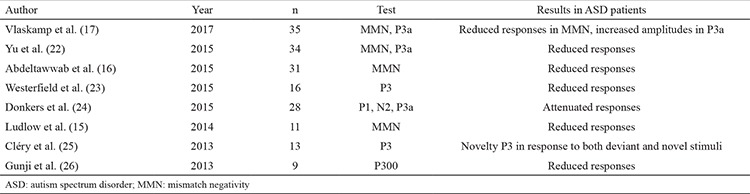
Results of recent studies using electrophysiological tests in patients with autism spectrum disorders
